# Behavioural stochastic resonance across the lifespan

**DOI:** 10.3758/s13415-024-01220-w

**Published:** 2024-09-10

**Authors:** Michele Di Ponzio, Luca Battaglini, Marco Bertamini, Giulio Contemori

**Affiliations:** 1 Neuro-X Institute, École Polytechnique Fédérale de Lausanne, Geneva, Switzerland; 2 Department of General Psychology, University of Padova, Padua, Italy; 3 Neuro.Vis.U.S. Laboratory, University of Padova, Padua, Italy; 4 Centro Di Ateneo Dei Servizi Clinici Universitari Psicologici (SCUP), University of Padova, Padua, Italy

**Keywords:** Stochastic resonance, Neural noise, Visual perception, Ageing, Psychophysics

## Abstract

Stochastic resonance (SR) is the phenomenon wherein the introduction of a suitable level of noise enhances the detection of subthreshold signals in non linear systems. It manifests across various physical and biological systems, including the human brain. Psychophysical experiments have confirmed the behavioural impact of stochastic resonance on auditory, somatic, and visual perception. Aging renders the brain more susceptible to noise, possibly causing differences in the  SR phenomenon between young and elderly individuals. This study investigates the impact of noise on motion detection accuracy throughout the lifespan, with 214 participants ranging in age from 18 to 82. Our objective was to determine the optimal noise level to induce an SR-like response in both young and old populations. Consistent with existing literature, our findings reveal a diminishing advantage with age, indicating that the efficacy of noise addition progressively diminishes. Additionally, as individuals age, peak performance is achieved with lower levels of noise. This study provides the first insight into how SR changes across the lifespan of healthy adults and establishes a foundation for understanding the pathological alterations in perceptual processes associated with aging.

## Introduction

Surprisingly, introducing a specific amount of noise to a subthreshold stimulus can improve signal detectability—a phenomenon known as stochastic resonance (SR) (McDonnell & Abbott, 2009; Moss & Wiesenfeld, 1995; Moss et al., 2004). One practical application of SR is observed in image restoration, where controlled, typically low-level noise is strategically introduced to a degraded image. This carefully calibrated noise interacts with existing image information, serving as a catalyst to amplify weak or subtle features (Kojima et al., 2019).

In investigating SR-like phenomena through behavioural tasks, plotting individual performance against external noise levels reveals a characteristic inverted U-shaped function (McDonnell & Abbott, 2009), indicating optimal performance at an intermediate level of noise (Moss et al., 2004; Simonotto et al., 1997). Concerning the mechanism at the basis of SR, the energy of the noise acts like a pedestal (Moss et al., 2004). Consequently, in the absence of noise, the energy of the stimulus is insufficient for detection. By adding the optimal amount of noise, the total energy (signal + noise) rises above the threshold and the stimulus becomes detectable. Conversely, when the noise is too high, it overwhelms the signal, causing a degradation in performance. When the amount of noise is on the sweet spot, the peak performance is achieved (van der Groen et al., 2018).

The term SR, initially introduced in the realm of physics, has garnered attention across various perceptual domains. For instance, in the sensorimotor domain, Mendez-Balbuena et al. (2012) illustrated that applying mechanical noise at varying levels to participants’ arms enhanced motor output at moderate noise levels. Similarly, Zeng et al. (2000) demonstrated auditory SR by using white noise to aid speech perception in hearing-impaired individuals, finding that specific noise intensities improved recognition capabilities, thereby supporting the application of SR in auditory assistive technologies. In tactile domains, Collins et al. (1996) manipulated mechanical noise intensities applied to the fingertip, which enhanced the detection of weak tactile stimuli. Vestibular SR was examined by Iwasaki et al. (2014) and Mulavara et al. (2011), who found that noise could enhance postural stability, suggesting potential applications in balance disorder rehabilitation.

Visual SR has been extensively investigated through various methods, including computational modelling, laboratory experiments, and brain stimulation techniques (Kim et al., 2006; van Boxtel, 2019; Ward et al., 2002, 2006; Yamazaki & Lioumis, 2022). Simonotto et al. (1997) showed that an appropriate amount of noise (gaussian noise) led to improvements in contrast detection of a digitized picture of Big Ben. Runnova et al. (2016) introduced the concept of “effective noise intensity” to quantify brain activity during the perception of ambiguous images, such as the Necker cube, confirming that optimal noise levels could indeed enhance visual perception. Studies by Kundu and Sarkar (2015) and Sasaki et al. (2006) focused on contrast sensitivity enhancements due to SR, whereas Treviño et al. (2016) and Itzcovich et al. (2017) expanded this research to motion perception and clinical populations, respectively. Itzcovich et al. (2017) found that noise could assist in motion perception tasks for individuals with visual impairments.

In the field of neuroscience, an ongoing debate surrounds the question of whether the brain has evolved to incorporate random noise in vivo as part of the “neural code.” This idea is supported by indirect evidence of a positive role for noise in the brain (McDonnell & Abbott, 2009). However, this hypothesis is complicated by the existence of various sources of noise that interact together. For instance, *external noise* includes irrelevant perceptual stimuli, whereas *neural noise* encompasses random fluctuations in brain activity at both the single cell level and the neural network level (Dinstein et al., 2015). At the cellular level, sources include electrical noise, thermal agitation, channel noise, neural adaptation, and synaptic plasticity (Clifford et al., 2007; Faisal et al., 2008; Feldman, 2009; Manwani & Koch, 1999). At the network level, random fluctuations arises from homeostatic adjustments to excitation/inhibition balance, changes in attention and arousal, interactions among large neural populations, and modulation of inter-regional connections (Clare Kelly et al., 2008; Fontanini & Katz, 2008; Turrigiano, 2011; van den Brink et al., 2019). These combined mechanisms (and others) may generate substantial variability both at rest and across trials during a task performance (Baracchini et al., 2021; Uddin, 2020).

A few studies have investigated the combined influence of external and neural noise in eliciting SR-like phenomena and found that that external and neural noise could synergistically enhance sensory and cognitive functions. Kitajo et al., (2003, 2007) found that introducing visual noise to one eye could improve visual signal detection in the other, an effect correlated with increased brain-wide phase synchronization. Aihara et al. (2008) explored visual detection in the presence and absence of visual noise, defining indices to measure external stochastic resonance effects and their correlation with internal noise levels, which was supported further by computational models (Aihara et al., 2010).

Recent research has shifted toward modulating internal neural noise through electrical stimulation rather than external noise sources. This research includes studies by Battaglini et al. (2023), Pavan et al. (2019), and van der Groen et al., (2018; 2016), who employed noninvasive transcranial random noise stimulation (tRNS) to modulate neural noise in targeted brain areas responsible for visual processing.

In addition to immediate changes, neural noise is subject to long-term changes that take place throughout life. In the process of healthy aging, the brain undergoes gradual structural changes while maintaining a high level of cognitive ability, in the sense that these changes often are accompanied by a reorganization of functional brain networks (Reuter-Lorenz & Park, 2014). Various neurocognitive theories of aging have debated whether such changes are advantageous or detrimental. Some argue that age-related changes are due to the combined effects of age-related structural changes and the change in the dynamics of a metastable system (Naik et al., 2017).

The neural noise hypothesis of aging suggests that owing to age-related increased neural noise, the signal-to-noise ratio (SNR) of the central nervous system degrades during aging, which leads to diminished cognitive performance (Cremer & Zeef, 1987; Li et al., 2001; Welford, 1981). In support of this hypothesis, Voytek et al. (2015) have measured with EEG the within-population spiking asynchrony and the local field potential 1/f slope. They observed an age-related flattening in the EEG 1/f slope, which means a higher level of 1/f noise. These changes have been associated with cognitive decline, specifically with working memory (Voytek et al., 2015). Further findings confirmed that with age there is a notable flattening of the 1/f exponent, indicative of an increase in local asynchronous activity in the cortex (Cesnaite et al., 2023; Clark et al., 2024; Dave et al., 2018; Finley et al., 2024; Merkin et al., 2023; Pathania et al., 2022; Tran et al., 2020; Voytek et al., 2015; Waschke et al., 2017), which is associated with cognitive decline in specific areas, such as working memory (Thuwal et al., 2021; Voytek et al., 2015), speeded processing (Pathania et al., 2022), and visual spatial attention (Tran et al., 2020).

Another line of research has instead suggested that aging is associated with a reduction in neural noise, reporting a decrease in trial-to-trial variability in BOLD responses and a consequent behavioural decline (Garrett et al., 2011). Variability has been demonstrated to convey task-related information in animal models (Denfield et al., 2018; Scaglione et al., 2011) and in human studies (Burlingham et al., 2022; Dinstein et al., 2015). In this sense, neural variability is a positive, not negative, factor (Waschke et al., 2021), indicative of neural adaptability to tasks (Nomi et al., 2017). Beyond the conceptualization of variability as noise, a deeper analysis of Garrett’s results (2011) reveals that only some brain areas have been found to show less variability in older people. In contrast, other areas have shown the opposite pattern. Moreover, a key finding was that the pattern of variability in the aged brain was more diffuse. This last finding can be not a hallmark of a reduction in neural noise, but can result from age-related dedifferentiation. Furthermore, measuring EEG activity, P300 latencies (reflecting cognitive functions) appeared to be more variable with age (Kilgler et al., 1993). However, variability in BOLD and EEG signals at rest relates differently to aging in the human brain (Kumral et al., 2020), partially owing to the different temporal resolutions of these tools. Given the temporal resolution, fMRI signal fluctuations are the result of time integration of activation across large neural populations and therefore are indices of network-level variability connected to structural brain connectivity (Baracchini et al., 2021; Fallon et al., 2020). Although EEG predominantly reflects oscillatory activities, it also is influenced by microscale neurophysiological factors; aperiodic neural activity can generate detectable scalp potentials and shape broadband EEG features (Brake et al., 2024). Background aperiodic or “scale-free” broadband activity is present across all frequencies, adhering to a 1/f power distribution where spectral power decreases with increasing frequency (Pritchard, 1992), constituting a large proportion of the spontaneous neural activity recorded from the cortex (He et al., 2010). 1/f spectral exponent has been shown to track variations in the balance of excitation and inhibition (E/I; Gao et al., 2017), which in turn is closely linked to neural variability on fine timescales (Harris & Thiele, 2011; Kanashiro et al., 2017). Therefore, it is important to note that the choice and interpretation of variability measures strongly affect the direction of observed effects (Waschke et al., 2021).

Mechanistically, increased neural noise is attributed to a dysregulation of homeostatic protective mechanisms in late adulthood, altering the inhibition/excitation balance (Radulescu et al., 2023). Ageing induces an increase in excitatory signalling (Rozycka & Liguz-Lecznar, 2017), causing an E/I imbalance, which then increases internal noise levels (Bennett et al., 2007; Casco et al., 2017; Cremer & Zeef, 1987; Hua et al., 2006; Tran et al., 2020; Voytek et al., 2015; Welford, 1981), primarily due to a reduction of GABA (Chamberlain et al., 2021; Hickmott & Dinse, 2012; Leventhal et al., 2003). In addition to the neurochemical alterations, the SNR during visual perceptual processing in the senescent brain is compromised by a reduction in structural integrity that triggers compensatory mechanisms in aged visual circuits (Parvez et al., 2021; Silva et al., 2020). As a result, the effectiveness/occurrence of SR in the elderly might be altered (Li et al., 2006). In our study, we define neural noise as the asynchronous, randomly generated brain activity that constitutes the aperiodic component of the EEG spectrum. Because the optimal functioning of SR requires an optimal overall sum of internal and external noise, alterations in internal noise levels necessitate adjustments to external noise to maintain the appropriate overall noise level (Aihara et al., 2008; Yi et al., 2006; Zhang et al., 2017).

A recent study by Battaglini et al. (2023) supports this interpretation. They stimulated the cortex of healthy adults with a noninvasive neuromodulatory technique based on an alternating current with randomly varying intensity (tRNS), which was supposed to modulate the amount of neural noise. Participants performed a coherent motion detection task in which external noise was manipulated by varying the total number of dots. Results showed that neuromodulation induced by tRNS caused a leftward shift in the peak of performance when accuracy was plotted as a function of external noise (dots numerosity). More specifically, when 1 mA-tRNS was applied to increase neural noise, the peak in performance was obtained for a lower level of external noise. This result suggests an inverse relationship between the amount of neural and external noise required to reach the threshold, and hence SR. In contrast, the results of the previous computational study by Li et al. (2006) suggested a direct relationship between the amount of neural^1^ and external noise required for optimal SR. In their study, a stochastic gain-tuning model was used to simulate changes in SR in the senescent brain. They reproduced the age-related increase in endogenous noise by changing the gain parameter (G) in the model. In doing so, they demonstrated that cognitive aging, characterized by heightened intrinsic neuronal noise but diminished plasticity, necessitates an increased amount of external noise for the SR phenomenon to manifest. This was illustrated by a consistent shift in peak performance towards higher levels of external noise, i.e., to the right in the inverted U-shaped curve (Li et al., 2006).

Given the conflicting evidence in the literature, understanding how age-related increases in neural noise impact the strength and occurrence of SR throughout the lifespan remains unclear. In our study, we aimed to test the predictions of Li et al. (2006) model by using psychophysical methods, focusing on age-related changes in SR within the adult visual system.

Motion perception is an ideal model for studying perceptual aging (Billino & Pilz, 2019). We employed the Random-Dot Kinematogram (RDK) task, where participants detect coherent motion amidst randomly moving dots. This task requires integrating multiple motion signals for accurate detection. Ho and Giaschi (2009) demonstrated that both low- and high-level versions of first-order RDKs activate motion-sensitive areas, such as MT, indicating their role in processing varying complexities of motion information. Moreover, coherence perception correlates directly with activity levels in the motion-sensitive middle temporal area MT. Previous studies have shown that age-related changes impact both motion perception thresholds and activity in motion-sensitive brain regions (Biehl et al., 2017; Ward et al., 2018).

Our study involved a group of healthy adults spanning an age range from 18 to 82 years, all of whom engaged in a two-interval forced-choice task. Participants were required to determine which of two successive displays contained coherent motion. This setup allowed us to explore age-related changes in SR using two distinct experimental blocks, each with specific manipulations. In the initial block, we manipulated the coherence level of the stimuli. Coherence level in this context refers to the ratio of dots moving coherently in a specified direction (e.g., rightward) to the total number of dots presented. In contrast, the second block kept coherence level constant while varying dot density. This manipulation involved adjusting the total number of dots within the same spatial area of the display, thereby increasing perceptual noise in the stimulus.

Stochastic resonance occurs with stimuli close to the perceptual threshold. The initial block also served to calibrate task difficulty through an adaptive procedure, aiming for 70.7% accuracy based on individual coherence thresholds. By standardizing task difficulty across participants in this way, we could later isolate the effects of external noise (dot density) on SR from individual differences in coherence thresholds.

It is crucial to distinguish between manipulating dot density and manipulating coherence level or SNR, typically defined as the ratio of coherent dots moving rightward to randomly moving dots. Increasing dot density introduces Correspondence Noise, stemming from uncertainties in matching dots across frames, which may mask coherent motion (Barlow & Tripathy, 1997; Tripathy et al., 2012). A large increase in density not only activates occipital areas more but also tends to reduce task accuracy (Ho & Giaschi, 2009; Tripathy et al., 2012). We expect increased external noise due to increased density to interact with internal noise.

In both blocks, the coherent dots were consistently present on one of the two screens per trial, although participants often erred. An incorrect response indicates that the coherent dot signal failed to exceed the participant's detection threshold. Adding noise (increasing density) that results in fewer errors (increased accuracy) implies that the noise raised the signal above the detection threshold by enhancing detector aggregation (integrative mechanisms). Conversely, if performance deteriorates, it suggests that the noise has likely overwhelmed the signal, consistent with the “classical” threshold SR paradigm.

Participants in the thresholding block were randomly assigned to two subgroups tested with different dot numbers (100 vs. 400 dots). This division served dual purposes. First, it ensured that participants did not exhibit better performance in the second block under the same density conditions as in the first block due to practice effects. By employing two densities for the staircase, we could detect an eventual bimodal distribution in the second block indicative of practice effects, independent of age. Second, it enabled us to explore the impact of density on coherence level thresholds across different age groups by comparing the slope of age-related threshold regressions between the 100-dot and 400-dot groups.

In the thresholding block, we expected a general age-related deterioration in performance and better thresholds in the condition with fewer dots (less external noise) for older adults (Hutchinson et al., 2014). For SR in the constant stimuli block, we expected to observe the typical peak at intermediate levels of external noise in young adults and a flattening of the curve with aging (Li et al., 2006). Regarding the positioning of the peak with respect to the amount of noise, we hypothesized two possible outcomes: 1) as age increases, the peak shifts to the left, i.e., inverse relationship between neural and external noise (Battaglini et al., 2023); and 2) as age increases, the peak shifts to the right, i.e., direct relationship between neural and external noise (Li et al., 2006).

## Methods

### Participants

A total of 286 participants took part in the experiment, all possessing normal or corrected-to-normal vision. The mean age of the participants was 45.92 years (SD = 19.85). Before the commencement of the study, three exclusion criteria were established:

1. Participants with a coherent motion threshold above 75% in the initial block staircase were excluded (32 participants).
2. Participants who did not achieve a minimum of 6 reversals in the last 40 trials of the first block, which is necessary to ensure accurate threshold estimation, were excluded (5 participants).
3. Participants with an average percentage of correct responses exceeding 90% in the second block were excluded to avoid ceiling effects (28 participants).

**Fig. 1:**
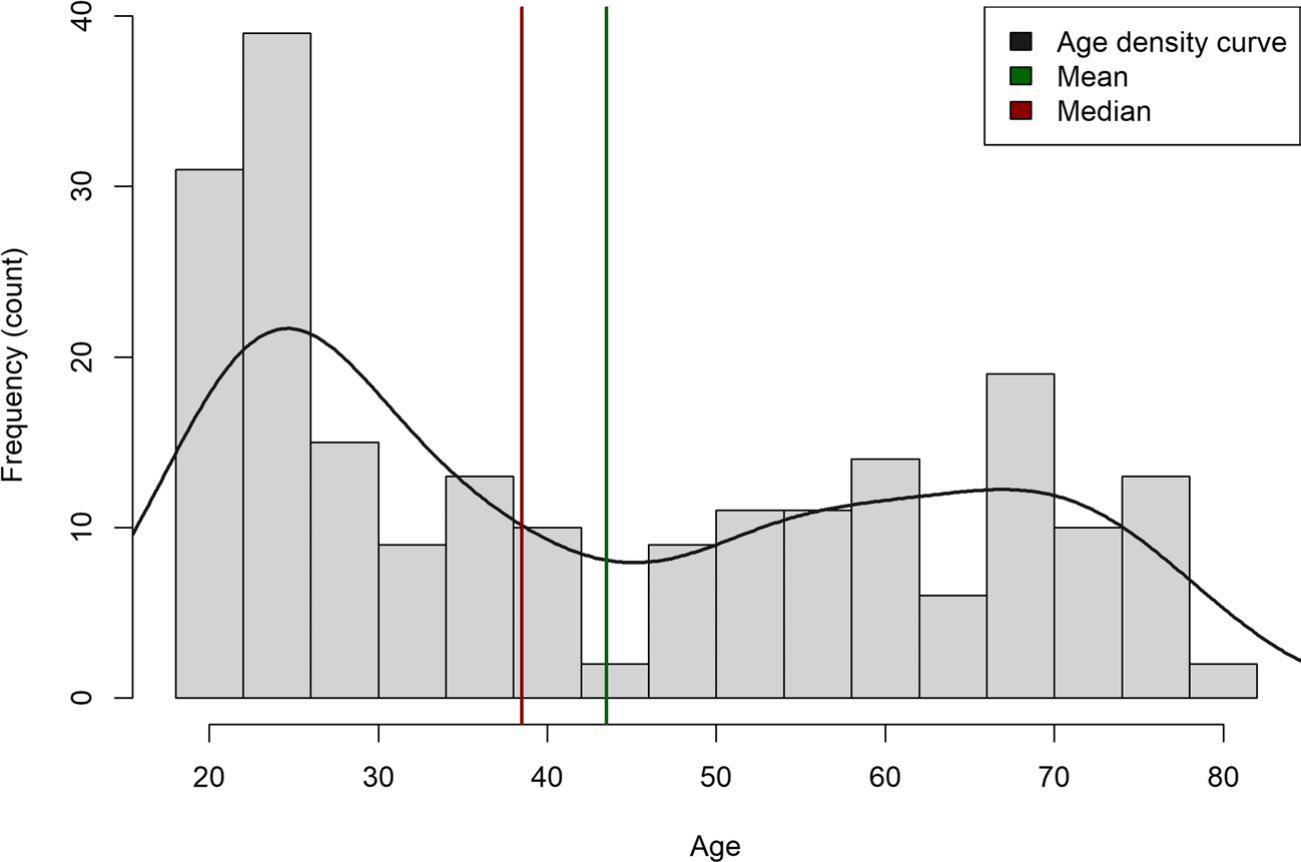
**Frequency distribution of the age of the participants.** Histogram represents the frequency distribution of age in the dataset, with a density curve (grey line) overlaid. Green line indicates the mean age, while the red line represents the median age. Bin width for the histogram is set to 4 units. The plot provides insights into the central tendency and distribution of age in the examined population

After applying these exclusion criteria, 214 participants remained for subsequent analysis. This final sample included 92 females and had a mean age of 43.52 years (SD = 19.61), with the age range spanning from 18 to 82 years. The age distribution of the final sample is depicted in Fig. 1.

The Ethics Committee of Psychological Research (Area 17) at the University of Padova granted approval for the experiment (protocol 4014). The procedures employed in this study align with the principles outlined in the Declaration of Helsinki. Before data collection, all participants provided informed consent for both participation in the study and the publication of anonymized aggregated data.

### Apparatus and stimuli

Participants performed the task on their own computer, remotely via the internet. At the beginning of the experiment, they were asked to sit in a quiet, semidark room, with no direct light on the screen. The task was implemented using HTML (Hyper Text Markup Language), CSS (Cascading Style Sheets) and jsPsych, an open-source JavaScript library developed specifically as a framework for web-based psychological experiments (de Leeuw, 2015). The experiment was hosted on a server located at the Department of General Psychology at the university of Padova and was made available online thanks to a JATOS instance (Lange et al., 2015). Participants were recruited among the acquaintances of the experimenters' collaborators and were unaware of the goals of the study. Each participant received the link for participation in the experiment via email together with a copy of the informed consent. A computer with a screen size of at least 10 in. was required for participation. People with touchscreen devices, such as smartphones and tablets, were excluded from participation. Participants were also asked to maintain a viewing distance of 57 cm from the screen, with the monitor perpendicular to the line of gaze throughout the experiment. The first screen presented participants with an informed consent form, before continuing. In a second screen, after entering some information (age, gender, and handedness), participants were asked the size of the monitor in centimetres. Based on the size of the monitor and resolution, we then calculated the individual number of pixels per degree of visual angle. Subsequently, all visual elements in the experiment were scaled according to this number to ensure consistency of stimulus size between participants.

**Fig. 2:**
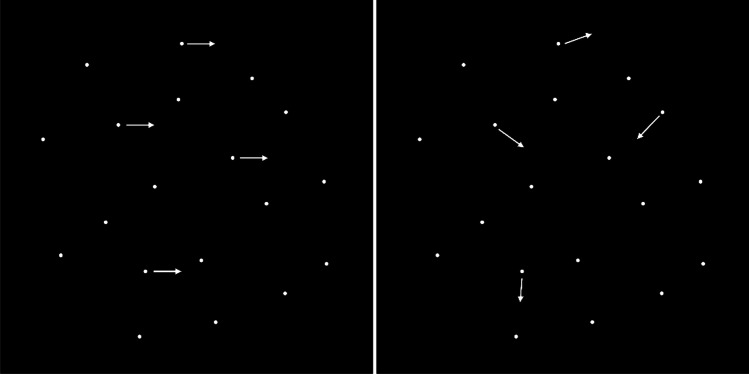
**Statically, two examples of the configuration of moving dots presented throughout the experiment.** A configuration of moving dots with a percentage of coherently moving dots (left). All the dots move randomly (right)

The Random Dot Kinematogram (RDK) is a stimulus widely used in motion studies both in lab and online (Rajananda et al., 2018). In RDKs, a certain percentage of dots are designated to move in one coherent direction, and the remaining percentage of dots are designated to move in random directions. In this experiment, coherent dots moved in the direction of coherent motion in all frames, contrary to random dots. The direction of the coherently moving dots was rightward; the other dots moved to an adjacent position in a random direction in each frame (“random walk”). The stimuli consisted of white dots moving on a black background (Fig. 2). The aperture was a square window of 10°, displayed at the centre of the screen while dot diameter was 0.075°. In each frame, dot displacement was 0.05°, resulting in a speed of 3 deg/s. The “dot lifespan,” which determines the time that pass before a dot disappears and reappears somewhere else within the aperture, was set to be longer than the stimulus duration. If a dot reached the end of the squared aperture, they were then reallocated to a random point on the opposite edge. Stimuli were created by means of the RDK jsPsych plugin (Rajananda et al., 2018).

### Procedure

**Fig. 3:**
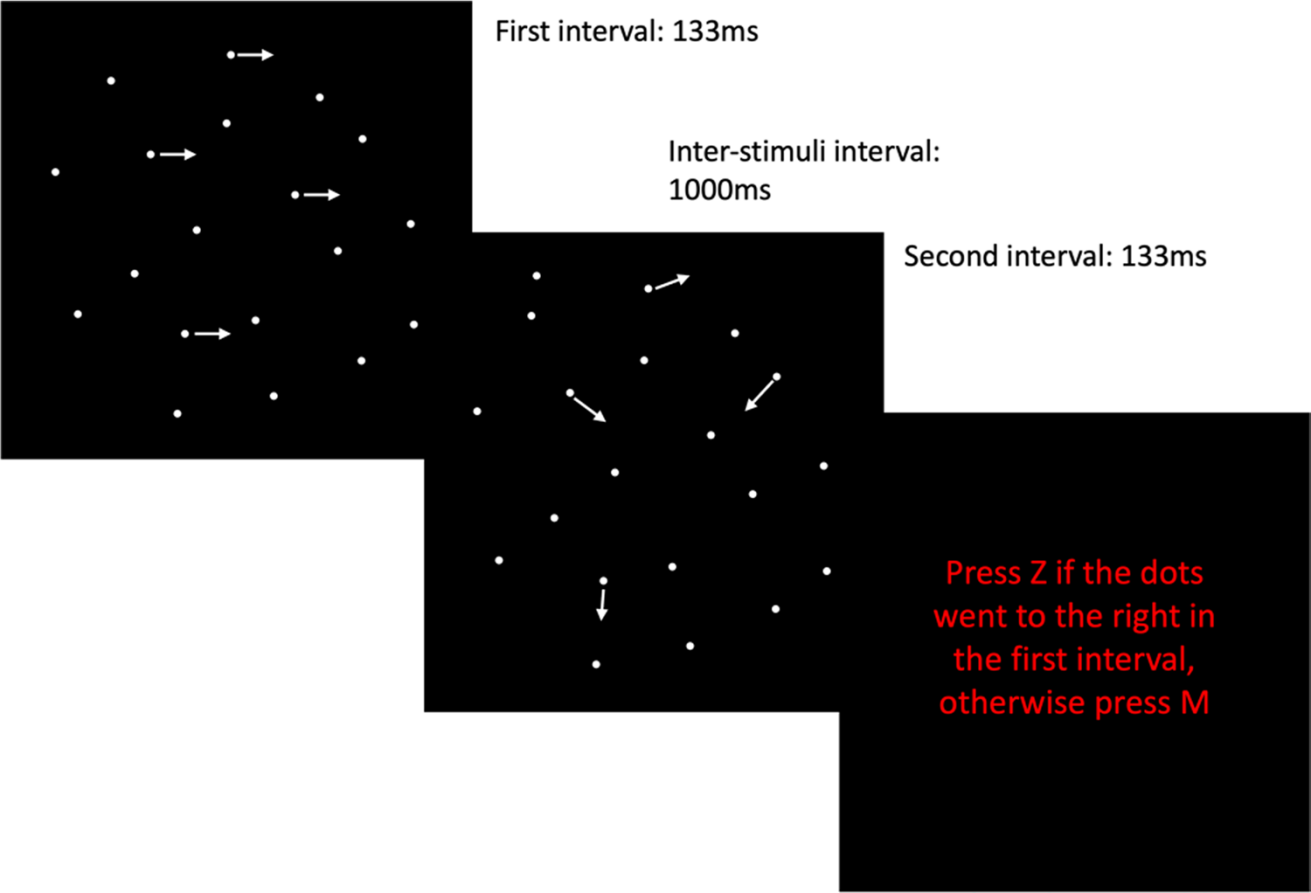
**Trial sequence consisted of a 133-ms initial interval featuring coherent (or random) motion, followed by a 1000-ms poststimulus gap.** Subsequently, there was a second 133-ms interval with random (or coherent) motion, another 1000-ms poststimulus gap and, finally, the participant's response. During the practice block, the response was followed by visual feedback. Trial-by-trial instructions were exclusively presented during practice trials. In the experimental trials, a red circle replaced the instructions, signalling participants when to provide their response

The motion detection task used a two-interval forced-choice paradigm and was mutuated from two previous laboratory studies in which an inverted U-shaped curve for performance as a function of dots numerosity was found (Battaglini et al., 2020, 2023). Participants decided which of two sequentially presented RDKs displayed a more coherent rightward direction of motion (Fig. 3).

The experiment comprised two blocks: a thresholding block and a constant stimuli block. Each of these blocks was preceded by a 10-trial practice session.

In the initial thresholding block, the total number of dots remained constant, while the proportion of coherently moving dots varied using a one-up two-down Levitt staircase (Levitt, 1971). Participants in this block were randomly assigned to one of two subgroups. One subgroup (N = 118, mean age = 43.18) had an initial threshold determined with a numerosity of 100 dots; the other subgroup (N = 96, mean age = 43.94) had a numerosity of 400 dots.

In the staircase procedure, the initial coherence level was set at 70%, and the initial step size was set at 10%. Following each reversal, the step size progressively decreased by 5%, 3%, 2%, and ultimately 1%. The staircase concluded after 12 reversals, and the threshold was computed as the average of the reversals, excluding the initial 4. This average denoted the minimum coherence level required for observers to accurately detect rightward motion 70.7% of the time.

Instructions were repeated before each trial, and feedback on response accuracy was provided. Each trial commenced with a fixation cross displayed for 1000 ms in red with a visual angle of half a degree. Subsequently, the first RDK was presented, followed by a 1000-ms poststimulus gap, and then the second RDK. Another 1000-ms poststimulus gap preceded participant response. A 100-ms warning sound preceded the presentation of both RDKs.

In each trial, a standard RDK consisted of randomly moving dots, alongside a target RDK containing a few coherent rightward moving dots (Fig. 3). Participants were instructed to press the "z" button if the target was in the first interval or the "m" button if in the second interval. Responses were recorded after both intervals were presented, and the subsequent trial began automatically upon pressing the response key. A red circle in experimental trials signalled when participants should respond.

The second block was identical to the first except for the change in the procedure from adaptive to constant stimuli. The random-to-coherent dots ratio was set based on the threshold obtained from the first block. In alignment with the methodology of Battaglini et al. (2023), the overall number of dots varied across 14 levels, strategically selected along a quasi-logarithmic scale (20, 29, 41, 58, 83, 118, 168, 239, 340, 485, 691, 999, 1403, and 2000 dots). The decision to adopt a logarithmic spacing was influenced by the understanding that the perception of motion and point numerosity adheres to a compressive nonlinearity, akin to Weber's Law (Zanker, 1995). Each of these 14 levels comprised 20 trials, resulting in a total of 280 pseudorandomized trials. Accuracy, defined as the proportion of correct responses at each noise level, was measured.

### Data analysis

We utilized the statistical software R (R Core Team, 2013) to conduct analyses and create figures. For the statistical analyses, continuous variables were first scaled and cantered using the “scale()” function in R.

In the first experimental block, our objective was to investigate the effects of dot density and age on coherence level thresholds. For this purpose, we employed a linear model with the natural logarithm (ln) of the threshold as the dependent variable. Coherence level thresholds are defined as the ratio of dots moving coherently in a specified direction (e.g., rightward) to the total number of dots presented, expressed as a percentage. The dependent variable ranged as an integer from 0 to 100, representing the percentage of coherent dots.

The choice of ln transformation aimed to capture the observed compressive nonlinearity in dot numerosity and motion perception (Zanker, 1995). This decision was reinforced by a substantial enhancement in the distribution of model residuals after the transformation. The model included the participant’s age as a continuous predictor and group as a two-level factor, representing the number of dots in the staircase procedure (either 100 or 400 dots). We assessed the significance of factors using an F-test.

For the examination of SR in the constant stimuli block, we employed a generalized linear mixed model with the binomial variable accuracy as the dependent variable. The model included the participant's age and the logarithm of the number of dots as continuous predictors. To address within-subject correlation in repeated measures, the model featured an individual random intercept. Regarding the number of dots, we hypothesized a nonlinear effect, resulting in an inverted U-shaped curve. Model selection involved comparing four mixed models, estimated using the glmer() function from the “lme4” package (Bates et al., 2019) in R. The number of dots variable was modelled with linear regression in one model and subsequently with polynomials up to the fourth degree. Orthogonal polynomials for the SOA variable were calculated using the poly() function in R.

We selected the best model based on the Akaike information criterion corrected for small sample sizes (AICc) with the aictab() function from the “AICcmodavg” package (Mazerolle, 2023). This test determined whether a nonmonotonic (curve) model outperformed a linear one. After choosing the best model, we conducted an omnibus test to assess significance, utilizing a Type III Wald chi-square tests with the Anova() function from the “CAR” package (Fox & Weisberg, 2019).

Given that the age distribution of participants (refer to Fig. 1) was nonuniform, during the review process, we accommodated a suggestion to incorporate a statistical test that would explore the effect of age as a binary factor rather than as a continuous variable. To implement this, we partitioned the sample into two age-based groups—“younger” individuals ranging from 18 to 50 years, and “older” individuals from 50 to 82 years, each spanning 32 years. Consequently, we adapted the previously selected optimal model by replacing the continuous age variable with a categorical variable representing these age groups. On this modified model, we again applied an omnibus test to assess significance using a Type III F-test with the Satterthwaite approximation for degrees of freedom.

We assessed model assumptions using the DHARMa R package (Hartig & Lohse, 2022). This package employs a simulation-based method to examine residuals for fitted Generalized Linear Mixed Models (GLMMs). The Asymptotic one-sample Kolmogorov–Smirnov test identified a significant deviation from the expected distribution for both the linear thresholding model (D = 0.05, *p* < 0.001) and the constant stimuli linear mixed model with both continuous (D = 0.033, *p* = 0.003) and categorical age (D = 0.032, *p* = 0.005). Upon visual inspection, this deviation appears negligible. Importantly, neither the dispersion test nor the outlier test yielded significant results. Therefore, in the absence of clear signs of heteroscedasticity or over/under dispersion, we proceeded with the analyses using these models.

For the LMM in the thresholding block results were reported using the “report” package (Makowski et al., 2020), and effect sizes were labelled following Field’s recommendations (Field, 2013). For the GLMM in the constant stimuli block as an estimate of the effect size, we calculated the semipartial coefficients of determination, also known as partR^2^ (∆R^2^), by means of the partR2 package (Stoffel et al., 2021). As suggested by Stoffel, part R2 for main effects and interactions were calculated separately and part R2 for the main effects were estimated after excluding the interaction from the model.

## Results

**Fig. 4:**
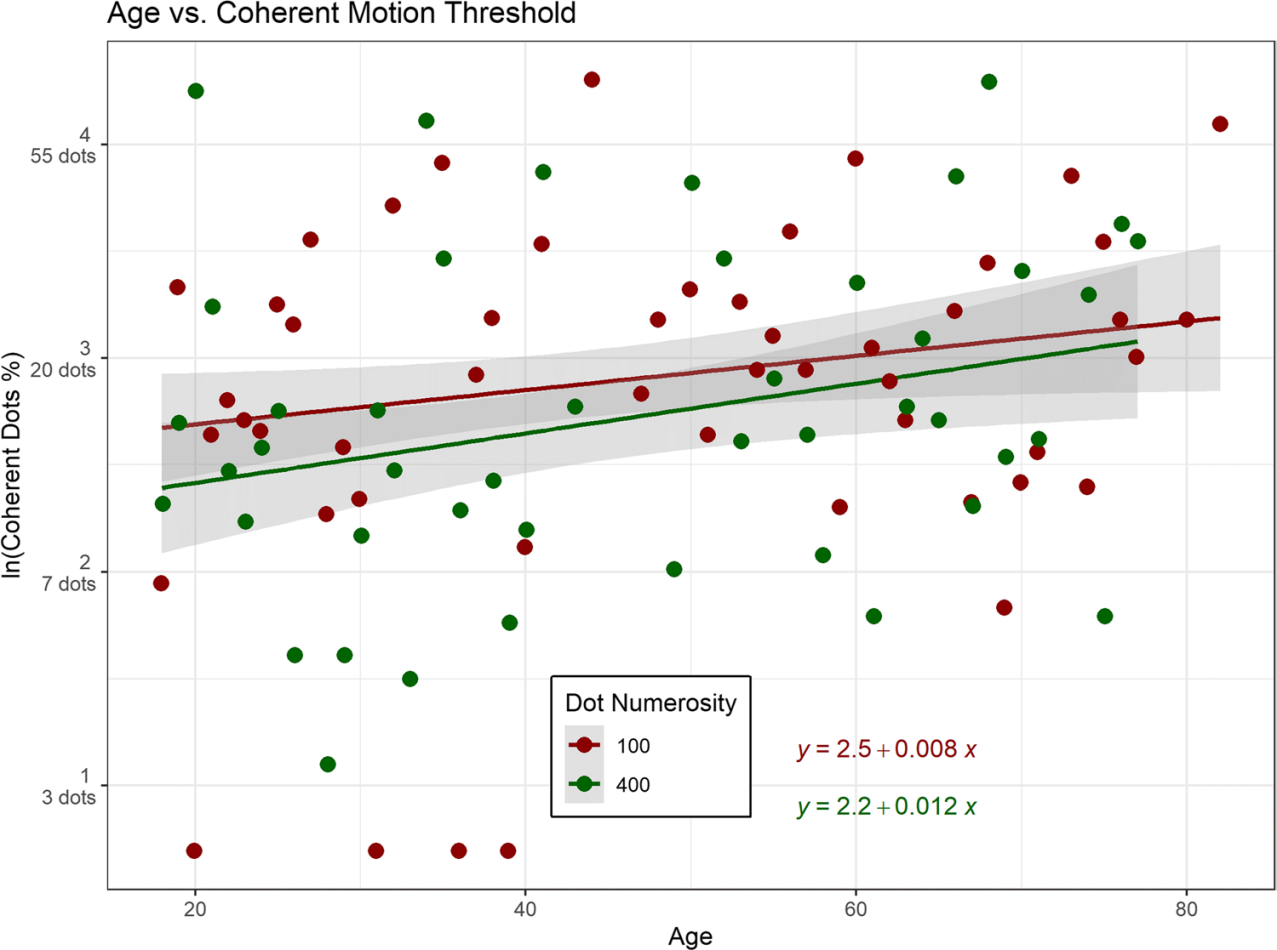
**Age-related changes in threshold for coherent RDK.** The plot shows how the ln(threshold) for detecting coherent motion, expressed as the natural logarithm of the percentage of coherent dots out of the total dots, varies with age for staircases with 100 and 400 dots. Linear regression lines, shaded 95% confidence bands, and individual data points are included. Red line corresponds to the staircase with 100 dots. Green line represents the staircase with 400 dots

In the initial experimental phase, we investigated the variation in performance, which we defined as the logarithm of the number of dots required to identify the interval with coherent motion 70.7% of the times. This exploration focused on the threshold for two levels of dot numerosity, specifically 100 and 400 (representing external noise), in relation to age. Figure 4 visually represents the ln(threshold) as a function of participants’ ages at two levels of dot numerosity.

The results, assessed through an F test, indicated a statistically significant yet small main effect of staircase dot numerosity (F(1, 2992) = 33.04, *p* < 0.001; partial η^2^ = 0.01, 95% CI [5.58e-03, 1.00]). The estimated marginal mean for the staircase with 100 dots was 2.88 (standard error = 0.021, degrees of freedom = 2992, CI [2.84, 2.92]), equivalent to 17.8 dots. In contrast, the estimated marginal mean for the staircase with 400 dots was 2.69 (standard error = 0.036, degrees of freedom = 2882, CI [2.64, 2.74]), equivalent to 14.7 dots.

The main effect of age was also statistically significant, though small (F(1, 2992) = 141.74, *p* < 0.001; Partial η^2^ = 0.05, 95% CI [0.03, 1.00]). With each passing year, there was an observed increase in the threshold of 0.192 on a logarithmic scale, equivalent to 1.21 dots.

Furthermore, the interaction between staircase dot numerosity and age was statistically significant, with a very small effect size (F(1, 2992) = 4.89, p = 0.027; Partial η^2^ = 1.63e-03, 95% CI [9.59e-05, 1.00]). For each year of age, the difference between the staircase with 100 dots and the one with 400 dots decreased by − 0.035 on a logarithmic scale, equivalent to − 0.96 dots.

**Fig. 5:**
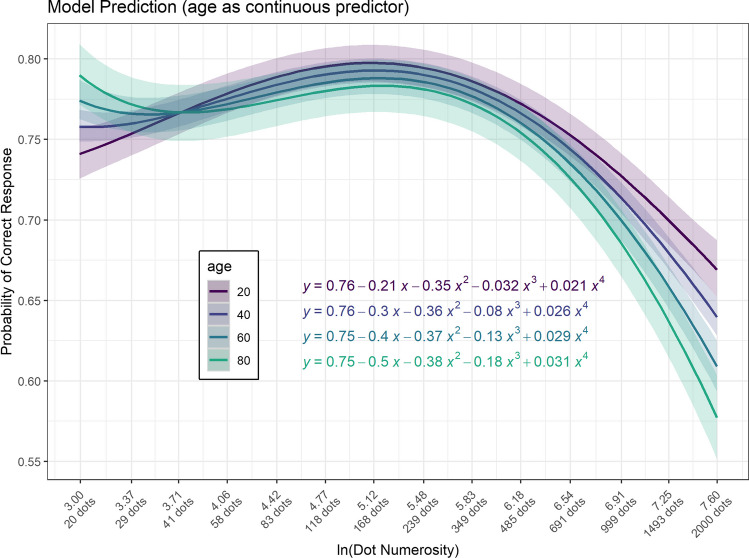
**The relationship between accuracy, dot numerosity, and age.** Lines represent predictions of the fourth-degree model. Although the age variable in the model is inherently continuous, for visualization, we have represented it through four age levels. The x-axis represents dot numerosity on a logarithmic scale. The coloured bands represent the 95% confidence intervals

When examining the relationship between performance and dot numerosity (Fig. 5), a peak was observed at intermediate levels. Visual exploration data were supported by model selection, where the model incorporating dot numerosity as a fourth-degree predictor outperformed other models (AICcWt = 0.64), particularly surpassing the linear dot numerosity model (AICcWt = 0). Comprehensive results of the model selection are presented in Table 1.

**Table 1:** Model selection based on AICc

Model	K	AICc	Delta AICc	AICcWt	Cum.Wt	LL
Fourth degree	11	13,408.62	0.00	0.64	0.64	− 6693.26
Cubic	9	13,409.76	1.14	0.36	1.00	− 6695.85
Quadratic	7	13,424.48	15.87	0.00	1.00	− 6705.22
Linear	5	13,720.58	311.96	0.00	1.00	− 6855.28

K = number of parameters in each model; AICc = Akaike Information Criterion corrected for small sample sizes; Delta AICc = difference in AICc values between each model and the best-fitting model; AICcWt = Akaike weight, representing the likelihood of each model being the best-fitting model; Cum.Wt = cumulative weight, the sum of AICc weights up to the current model; LL = log-likelihood, a measure of how well the model explains the observed data

The marginal R^2^ and 95% CI for the full model were R^2^ = 0.01, 95% CI [0.01, 0.013]. The omnibus Type III Wald chi-square test over the best model (fourth degree) suggested that the main effect of the logarithm of the number of dots was statistically significant and substantial, explaining a significant portion of the model's variance (χ^2^(4) = 611.2400, *p* < 0.001; ∆R^2^ = 0.01, 95% CI [0.008, 0.012]). The main effect of age was statistically not significant and very small (χ^2^(1) = 0.4579, *p* = 0.499; ∆R^2^ = 0.0002, 95% CI [0, 0.002]). The interaction between the logarithm of the number of dots and age was statistically significant and very small (χ^2^(4) = 28.7636, *p* < 0.001; ∆R^2^ = 0.0004, 95% CI [0, 0.001]).

Figure 5 illustrates the predictions generated by the fourth-degree model. Despite entering the age variable as a continuous parameter in the model, we opted to visually represent it across four distinct age levels. The model’s prediction for an individual aged 20 years is expressed by the equation $Y=0.76-0.21x-0.35{x}^{2}-0.032{x}^{3}+ 0.021{x}^{4}$, while for an 80-year-old individual, it is given by $Y=0.75-0.5x-0.38{x}^{2}-0.18{x}^{3}+0.031{x}^{4}$. It is noteworthy that with increasing age, there is a gradual augmentation in the negativity of both the linear and cubic terms. This interaction results in a flattening of the curve for lower dot numerosity values (left side of the curve) and a sharp decline in performance for higher dot numerosity (right side of the curve).

**Fig. 6:**
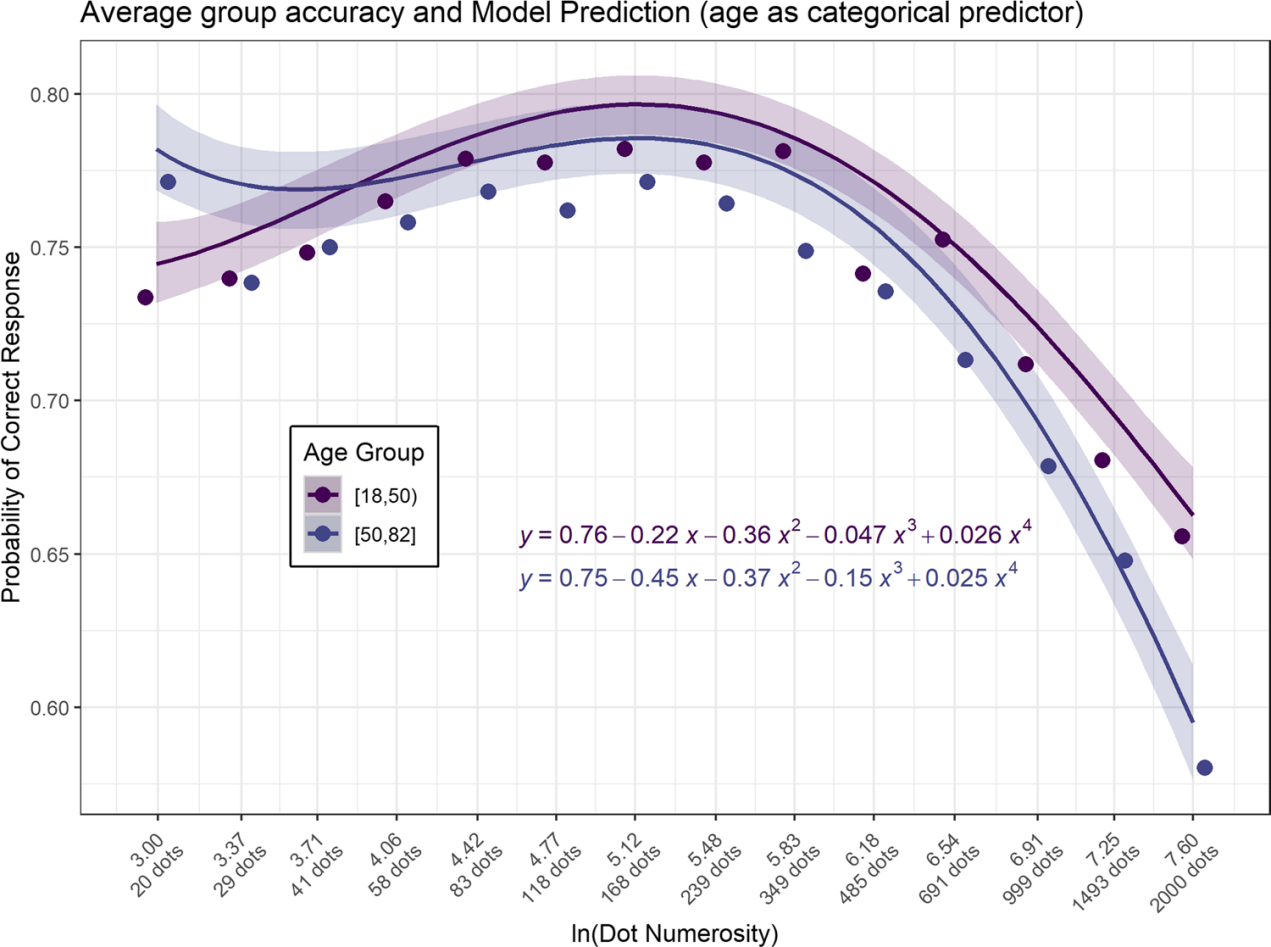
**The relationship between accuracy, dot numerosity, and age group as a categorical two-level factor.** Dots represent average accuracy for each group. Lines depict the predictions of the fourth-degree model. X-axis denotes dot numerosity on a logarithmic scale. Coloured bands represent the 95% confidence intervals

When analysing age as a dichotomous variable, the participants were divided into two age-defined groups: the younger group comprised 123 individuals within an age range of 18 to 50 years, and the older group included 91 individuals aged between 50 and 82 years. The marginal R^2^ and 95% CI for the full model were R^2^ = 0.01, 95% CI [0.01, 0.013]. The omnibus Type III Wald chi-square test indicated that the main effect of the logarithm of dot numerosity was statistically significant and substantial, explaining a significant portion of the model's variance (χ^2^(4) = 630.848, *p* < 0.001; ∆R^2^ = 0.01, 95% CI [0.008, 0.011]). Conversely, the main effect of age group did not reach statistical significance and had a negligible effect size (χ^2^(1) = 0.504, *p* = 0.478; ∆R^2^ = 0, 95% CI [0, 0.001]). However, the interaction between the logarithm of dot numerosity and age group was statistically significant, albeit small (χ^2^(3) = 38.031, *p* < 0.001; ∆R^2^ = 0.001, 95% CI [0, 0.003]). The predictive model for the younger group was described by the equation $Y = 0.76-0.22x-0.36- 0.047{x}^{3}+ 0.026{x}^{4}$, while for the older group, it was articulated as $Y=0.75-0.45x-0.37{x}^{2}-0.15{x}^{3}+ 0.025{x}^{4}$. The results further demonstrated an increase in the negative coefficients of linear and cubic terms with for the older age group, thus corroborating the findings obtained when age was modelled as a continuous variable. Figure 6 illustrates the predictions generated by the fourth-degree model with age group as a categorical two-level factor.

## Discussion

The purpose of the study was to investigate age-related changes in psychophysical stochastic resonance (SR) within human visual perception, considering external noise as a variable. A sample of healthy adults underwent a two-intervals forced-choice coherent motion discrimination task with RDK stimuli. Two separate blocks were administered. In the first, we determined the coherence level required for a 70.7% accuracy performance. In the second, using a constant stimulus procedure, we manipulated external noise (total dot number) to assess its impact on accuracy.

As expected, initial thresholding block indicated a decline in coherent motion detection ability with increasing participant age, aligning with findings from previous studies (Braham Chaouche et al., 2020; Pilz et al., 2017; Trick & Silverman, 1991) showing age-related deterioration in global motion perception. Coherent motion detection involves pooling similar motion signals while suppressing irrelevant ones (Dakin et al., 2005). This decline in ability may be attributed to increased tonic neural noise negatively affecting stimulus-associated neural variability in aging (Tran et al., 2020). Interestingly, younger participants demonstrated improved performance with more dots (400), whereas this advantage diminished for older adults. Higher dot numerosity theoretically facilitates global pooling by increasing the likelihood of combining motion signals from the target direction (Dakin et al., 2005). However, for older adults, the detrimental impact of increased global noise may outweigh the benefits of enhanced pooling.

Our results parallel findings by Hutchinson et al. (2014). In their investigation, they assessed the performance of both young and old participants under three distinct conditions: equal dot numbers but varied sizes and densities (exp. 1); uniform density but increased dot number and size (exp. 2); uniform size but heightened density and dot number (exp. 3). In contrast to younger participants, elderly participants in both Experiments 1 and 2 did not demonstrate improved performance as the dot number or size increased. However, in Experiment 3, mirroring our initial experimental phase, no statistically significant difference in performance between young and old participants was observed.

Despite the lack of statistical significance, a closer examination of the data revealed a notable trend—an inverse relationship between density and performance for both age groups. Young individuals excelled at high density, whereas older individuals exhibited better performance at low density. This observed trend in Hutchinson et al. (2014) is in agreement with our results in the thresholding block. The difference in statistical outcome may be attributed to a large difference in statistical power between the two studies (N = 18 in Hutchinson et al. compared with our N = 214). By integrating our results with prior research, we posit that in an aging neural system characterized by heightened intrinsic noise, performance is hindered rather than improved by increased dot number/density, unlike in younger individuals.

Following the standardization of baseline performance, we further examined the influence of advancing age on SR. Employing the constant stimulus method, we systematically varied the total number of dots from 20 to 2000 while maintaining a consistent stimulus size. Performance was then assessed across 14 levels of dot numerosity. As anticipated, the accuracy plotted against dot number exhibited the characteristic inverted U-shaped curve associated with SR (Battaglini et al., 2023).

This result was corroborated by model selection, wherein polynomial models of second degree and higher outperformed the linear model. The model offering the best balance between fit and parsimony, as determined by the AICc, was the fourth-degree polynomial model. However, the difference in AICc between the fourth-degree model and the cubic model was less than 2, indicating that both models were nearly equivalent, with a slight preference for the fourth-degree model owing to the decrease in log-likelihood (Arnold, 2010). Notably, the fourth-degree term exhibited minor age-related differences, whereas significant differences were observed in the linear and cubic terms, which became increasingly negative with advancing age.

According to the predictions of the fourth-degree model, as age increases, the curve becomes less steep to the left of the peak and steeper to the right. Surprisingly, in the oldest individuals (around 80 years of age), optimal performance was observed at the lowest external noise level (peak shifted to the left), with accuracy declining as the number of dots increased. This differs from the beneficial SR effect observed in younger participants. These findings suggest that with advancing age and increased neural noise, the sweet point for optimal performance shifts towards lower external noise levels.

Concerning our initial hypothesis, whereas the flattened curve in the elderly aligns with predictions from Li & colleagues (2006), the leftward shift of optimal performance does not. Instead, it aligns with the findings of Battaglini et al. (2023). For younger adults, the addition of weak external noise may not surpass the threshold, resulting in suboptimal performance. Conversely, in the brains of older adults, the weak external noise combines with an elevated level of internal noise, potentially triggering SR. When external noise is optimized for inducing SR in younger individuals, performance deterioration is observed in older individuals.

The addition of an optimal level of external noise has been demonstrated to amplify responsiveness to visual stimuli by boosting the neuronal firing rate (Srebro & Malladi, 1999). However, in the elderly, the baseline noise in the visual cortex is already high (Talyansky & Brinkman, 2021; Tran et al., 2020; Yan et al., 2020). Consequently, lower amounts of external noise may be sufficient to trigger SR in older adults due to the combination with heightened levels of internal neural noise.

Similar interaction between external and internal noises has been previously reported both in psychophysics and electrophysiology (Aihara et al., 2010; Douglass et al., 1993). For example, Douglass et al. (1993) found higher-than-expected detection accuracy values in single sensory neurons of crayfish at low external noise levels, proposing that neural noise played a role in this phenomenon. Additionally, Varlet & Richardson (2016) demonstrated a mutual relationship between internal and external noise for neural synchrony, indicating that moderate internal noise facilitates synchronization at minimal external noise levels, whereas high levels of both internal and external noise degrade synchronization.

Considering this theoretical background, our research supports theories suggesting nonlinear effects of neural noise changes on behaviour in noisy environments (Cremer & Zeef, 1987; Li et al., 2001; Welford, 1981), indicating that the effect of increased neural noise, which is marked by enhanced local neural excitability, as evidenced by a decrease in the 1/f exponent, also should be balanced on the levels of external noise.

Research by Tran et al. (2020) demonstrated that older adults with flatter spectral slopes exhibited more variability within the occipital alpha band, as measured by intertrial phase coherence, indicating an inverse relationship between an increase in neural noise (as a reduction in the exponent of the aperiodic component) and neural variability in response to stimuli. Furthermore, recent findings by Manyukhina et al. (2024) demonstrated a negative correlation between changes in periodic power and the aperiodic exponent observed before and after a task. This suggests that as the aperiodic exponent decreases (indicating an increase in tonic basal neural noise), there is a corresponding decrease in periodic power.

This interaction implies that heightened neural noise is associated with reduced neural responsiveness at the network level. Additionally, the study highlights a relationship between the aperiodic exponent and poststimulus neural inhibition in visual areas, suggesting that increased neural noise disrupts normal inhibitory processes, leading to altered neural responses following a stimulus. Therefore, an increase in noise at high frequencies of the spectrum appears to coincide with a disrupted homeostatic balance tipped in favour of excitation, leading to a loss of neural tuning.

According to Rubenstein and Merzenich (2003), cortical noise reflects a hyperexcitable and poorly differentiated cortex. Our findings also align with the Loss of Complexity in Aging Hypothesis (LOCH). Initially, when LOCH was introduced, complexity was defined as the extent to which the underlying system generates aperiodic fluctuations resembling nonlinear chaos. Complexity characterizes a state between order and disorder, where increasing randomness could lead to either an increase or decrease in complexity (Goldberger et al., 2002; Lipsitz & Goldberger, 1992). However, both a reduction in synchronized neural response variability and the increase in aperiodic basal noise could induce a loss of complexity, ultimately leading to decreased behavioural performance (Sleimen-Malkoun et al., 2014). Our study also may represent a support to dedifferention theory, according to which age-related decline results from increases in neural noise that reduce the selectivity of neural representations (Reuterr-Lorenz & Park, 2014). Loss of complexity along with dedifferentiation constitute two intertwined facets of the same aging process (Sleimen-Malkoun et al., 2014).

We acknowledge that our conclusions regarding internal noise are preliminary, given that internal noise levels were not directly quantified in our study. Notably, at the beginning of data collection, despite the pandemic abating, it remained inadvisable to involve elderly participants in a laboratory environment. Opting for online testing over in-laboratory assessments allowed for a broader age range, and thus, we employed age as a proxy for increased neural noise. Using age as a proxy for increased neural noise gains support from consistent findings across various independent studies that report a change in exponent with advancing age, underscoring the robustness of this assumption in our research framework. Collectively, these studies suggest a flattening—or reduction—in the exponent with age (Cesnaite et al., 2023; Clark et al., 2024; Dave et al., 2018; Finley et al., 2024; Merkin et al., 2023; Pathania et al., 2022; Tran et al., 2020; Voytek et al., 2015; Waschke et al., 2017), an event linked with an change in the E/I balance (Gao et al., 2017) and an increment in noise at higher frequencies, denoting more local (nonnetwork-level) and asynchronous cortical activity (Thuwal et al., 2021). The recent expansion in such research has been facilitated by the availability of numerous free toolboxes (Donoghue et al., 2020; Hu et al., 2024; Wen & Liu, 2016; Whitten et al., 2011; Wilson et al., 2022), which are integrated into user-friendly analysis platforms. These tools enable straightforward calculations of the exponent and intercept of the aperiodic portion of the EEG spectrum, further applied to investigate the impact of age on basal neural noise. Another limitation is the nonuniform age distribution in our sample. However, it is reassuring that similar results were observed when analysing age both as a continuous variable, as planned, and as a two-level factor. Lastly, we cannot fully exclude the possibility that differences in hardware typical of online data collection could have impacted data quality, despite the large sample size and rigorous planning of our study.

Future studies could address this limitation, potentially extending and generalizing these results to other tasks, types of noise manipulation, and age cohorts. Furthermore, investigating changes in SR in children and individuals with autism spectrum disorders, characterized by increased neural noise (Dinstein et al., 2012; Milne, 2011; Rubenstein & Merzenich, 2003), would be of interest.

## Conclusions

This study examined age-related changes in psychophysical stochastic resonance in human visual perception, focusing on the impact of external noise. Results confirmed age-related declines in coherent motion detection, aligning with previous findings. Younger participants showed enhanced performance with increased dot numerosity, whereas older adults experienced diminishing advantages, indicating a complex interaction between age, external noise, and task demands.

The investigation into SR revealed an unexpected inverted U-shaped curve, with optimal performance for older individuals occurring at lower external noise levels. This challenges the initial hypothesis and aligns with recent findings by Battaglini et al. (2023). Integrating these results with the neural noise hypothesis of aging suggests that increased internal neural noise may contribute to observed patterns. The interplay between external and internal noise in triggering SR aligns with previous research.

While the reduced ability of older adults to filter external noise may contribute to performance decrements, the speculative nature of conclusions related to internal noise is emphasized because of the absence of direct measurement. Future research should address this limitation, exploring implications in various cognitive tasks and extending findings to diverse populations, such as children and individuals with autism spectrum disorders. This investigation provides valuable insights into nuanced dynamics of age-related changes in visual perception, paving the way for further exploration of the interplay between external and internal noise in the aging brain.

## Data Availability

Data and materials, comprising a demo version of the task, data collection code, and analysis code, are available on the OSF repository. The study was not preregistered.

## Footnotes

1 Neural noise is called internal noise in Li et al. (2006).

## References

[CR1] Aihara, T., Kitajo, K., Nozaki, D., & Yamamoto, Y. (2008). Internal noise determines external stochastic resonance in visual perception. *Vision Research,**48*(14), 1569–1573. 10.1016/j.visres.2008.04.022 18514251 10.1016/j.visres.2008.04.022

[CR2] Aihara, T., Kitajo, K., Nozaki, D., & Yamamoto, Y. (2010). How does stochastic resonance work within the human brain? – Psychophysics of internal and external noise. *Chemical Physics,**375*(2), 616–624. 10.1016/j.chemphys.2010.04.027

[CR3] Arnold, T. W. (2010). Uninformative Parameters and Model Selection Using Akaike’s Information Criterion. *Journal of Wildlife Management,**74*(6), 1175–1178. 10.2193/2009-367

[CR4] Baracchini, G., Mišić, B., Setton, R., Mwilambwe-Tshilobo, L., Girn, M., Nomi, J. S., Uddin, L. Q., Turner, G. R., & Spreng, R. N. (2021). Inter-regional BOLD signal variability is an organizational feature of functional brain networks. *NeuroImage,**237*, 118149. 10.1016/J.NEUROIMAGE.2021.118149 33991695 10.1016/j.neuroimage.2021.118149 PMC8970039

[CR5] Barlow, H., & Tripathy, S. P. (1997). Correspondence noise and signal pooling in the detection of coherent visual motion. *Journal of Neuroscience,**17*(20), 7954–7966. 10.1523/jneurosci.17-20-07954.1997 9315913 10.1523/JNEUROSCI.17-20-07954.1997 PMC6793893

[CR6] Bates, D., Maechler, M., Bolker, B., & Walker, S. (2019). *Linear Mixed-Effects Models using “Eigen” and S4: Package “lme4.”* 123.

[CR7] Battaglini, L., Casco, C., Fertonani, A., Miniussi, C., Di Ponzio, M., & Vicovaro, M. (2023). Noise in the brain: Transcranial random noise stimulation and perceptual noise act on a stochastic resonance-like mechanism. *European Journal of Neuroscience,**57*(12), 2097–2111. 10.1111/ejn.15965 36922400 10.1111/ejn.15965

[CR8] Battaglini, L., Mena, F., & Casco, C. (2020). Improving motion detection via anodal transcranial direct current stimulation. *Restorative Neurology and Neuroscience,**38*(5), 395–405. 10.3233/RNN-201050 33016896 10.3233/RNN-201050

[CR9] Bennett, P. J., Sekuler, R., & Sekuler, A. B. (2007). The effects of aging on motion detection and direction identification. *Vision Research,**47*(6), 799–809. 10.1016/j.visres.2007.01.001 17289106 10.1016/j.visres.2007.01.001

[CR10] Biehl, S. C., Andersen, M., Waiter, G. D., & Pilz, K. S. (2017). Neural changes related to motion processing in healthy aging. *Neurobiology of Aging,**57*, 162–169. 10.1016/j.neurobiolaging.2017.05.018 28648917 10.1016/j.neurobiolaging.2017.05.018 PMC5538346

[CR11] Billino, J., & Pilz, K. S. (2019). Motion perception as a model for perceptual aging. *Journal of Vision,**19*(4), 3. 10.1167/19.4.3 30943529 10.1167/19.4.3

[CR12] Braham Chaouche, A., Silvestre, D., Trognon, A., Arleo, A., & Allard, R. (2020). Age-related decline in motion contrast sensitivity due to lower absorption rate of cones and calculation efficiency. *Scientific Reports,**10*(1), 16521. 10.1038/s41598-020-73322-7 33020552 10.1038/s41598-020-73322-7 PMC7536415

[CR13] Brake, N., Duc, F., Rokos, A., Arseneau, F., Shahiri, S., Khadra, A., & Plourde, G. (2024). A neurophysiological basis for aperiodic EEG and the background spectral trend. *Nature Communications 2024 15:1*, *15*(1), 1–15. 10.1038/s41467-024-45922-8 10.1038/s41467-024-45922-8 PMC10876973 38374047

[CR14] Burlingham, C. S., Ryoo, M., Roth, Z. N., Mirbagheri, S., Heeger, D. J., & Merriam, E. P. (2022). Task-related hemodynamic responses in human early visual cortex are modulated by task difficulty and behavioral performance. *ELife*, *11*. 10.7554/ELIFE.73018 10.7554/eLife.73018 PMC9049970 35389340

[CR15] Casco, C., Barollo, M., Contemori, G., & Battaglini, L. (2017). The effects of aging on orientation discrimination. *Frontiers in Aging Neuroscience*, *9*(MAR), 45. 10.3389/fnagi.2017.00045 10.3389/fnagi.2017.00045 PMC5332427 28303102

[CR16] Cesnaite, E., Steinfath, P., JamshidiIdaji, M., Stephani, T., Kumral, D., Haufe, S., Sander, C., Hensch, T., Hegerl, U., Riedel-Heller, S., Röhr, S., Schroeter, M. L., Witte, A. V., Villringer, A., & Nikulin, V. V. (2023). Alterations in rhythmic and non-rhythmic resting-state EEG activity and their link to cognition in older age. *NeuroImage,**268*, 119810. 10.1016/j.neuroimage.2022.119810 36587708 10.1016/j.neuroimage.2022.119810

[CR17] Chamberlain, J. D., Gagnon, H., Lalwani, P., Cassady, K. E., Simmonite, M., Seidler, R. D., Taylor, S. F., Weissman, D. H., Park, D. C., & Polk, T. A. (2021). GABA levels in ventral visual cortex decline with age and are associated with neural distinctiveness. *Neurobiology of Aging,**102*, 170–177. 10.1016/j.neurobiolaging.2021.02.013 33770531 10.1016/j.neurobiolaging.2021.02.013 PMC8205971

[CR18] Clare Kelly, A. M., Uddin, L. Q., Biswal, B. B., Castellanos, F. X., & Milham, M. P. (2008). Competition between functional brain networks mediates behavioral variability. *NeuroImage,**39*(1), 527–537. 10.1016/J.NEUROIMAGE.2007.08.008 17919929 10.1016/j.neuroimage.2007.08.008

[CR19] Clark, M., Euler, M. J., King, B. R., Williams, A. M., & Lohse, K. R. (2024). Associations between age-related differences in occipital alpha power and the broadband parameters of the EEG power spectrum: A cross-sectional cohort study. *International Journal of Psychophysiology,**195*, 112272. 10.1016/J.IJPSYCHO.2023.112272 38000446 10.1016/j.ijpsycho.2023.112272

[CR20] Clifford, C. W. G., Webster, M. A., Stanley, G. B., Stocker, A. A., Kohn, A., Sharpee, T. O., & Schwartz, O. (2007). Visual adaptation: Neural, psychological and computational aspects. *Vision Research,**47*(25), 3125–3131. 10.1016/J.VISRES.2007.08.023 17936871 10.1016/j.visres.2007.08.023

[CR21] Collins, J. J., Imhoff, T. T., & Grigg, P. (1996). Noise-enhanced tactile sensation. *Nature,**383*(6603), 770. 10.1038/383770a0 8893000 10.1038/383770a0

[CR22] Cremer, R., & Zeef, E. J. (1987). What Kind of Noise Increases With Age? *Journal of Gerontology,**42*(5), 515–518. 10.1093/geronj/42.5.515 3624810 10.1093/geronj/42.5.515

[CR23] Dakin, S. C., Mareschal, I., & Bex, P. J. (2005). Local and global limitations on direction integration assessed using equivalent noise analysis. *Vision Research,**45*(24), 3027–3049. 10.1016/j.visres.2005.07.037 16171844 10.1016/j.visres.2005.07.037

[CR24] Dave, S., Brothers, T. A., & Swaab, T. Y. (2018). 1/F Neural Noise and Electrophysiological Indices of Contextual Prediction in Aging. *Brain Research,**1691*, 34–43. 10.1016/j.brainres.2018.04.007 29679544 10.1016/j.brainres.2018.04.007 PMC5965691

[CR25] de Leeuw, J. R. (2015). jsPsych: A JavaScript library for creating behavioral experiments in a Web browser. *Behavior Research Methods,**47*(1), 1–12. 10.3758/s13428-014-0458-y 24683129 10.3758/s13428-014-0458-y

[CR26] Denfield, G. H., Ecker, A. S., Shinn, T. J., Bethge, M., & Tolias, A. S. (2018). Attentional fluctuations induce shared variability in macaque primary visual cortex. *Nature Communications 2018 9:1*, *9*(1), 1–14. 10.1038/s41467-018-05123-6 10.1038/s41467-018-05123-6 PMC6037755 29985411

[CR27] Dinstein, I., Heeger, D. J., & Behrmann, M. (2015). Neural variability: Friend or foe? *Trends in Cognitive Sciences,**19*(6), 322–328. 10.1016/j.tics.2015.04.005 25979849 10.1016/j.tics.2015.04.005

[CR28] Dinstein, I., Heeger, D. J., Lorenzi, L., Minshew, N. J., Malach, R., & Behrmann, M. (2012). Unreliable Evoked Responses in Autism. *Neuron,**75*(6), 981–991. 10.1016/j.neuron.2012.07.026 22998867 10.1016/j.neuron.2012.07.026 PMC3457023

[CR29] Donoghue, T., Haller, M., Peterson, E. J., Varma, P., Sebastian, P., Gao, R., Noto, T., Lara, A. H., Wallis, J. D., Knight, R. T., Shestyuk, A., & Voytek, B. (2020). Parameterizing neural power spectra into periodic and aperiodic components. *Nature Neuroscience,**23*(12), 1655–1665. 10.1038/s41593-020-00744-x 33230329 10.1038/s41593-020-00744-x PMC8106550

[CR30] Douglass, J. K., Wilkens, L., Pantazelou, E., & Moss, F. (1993). Noise enhancement of information transfer in crayfish mechanoreceptors by stochastic resonance. *Nature,**365*(6444), 337–340. 10.1038/365337a0 8377824 10.1038/365337a0

[CR31] Faisal, A. A., Selen, L. P. J., & Wolpert, D. M. (2008). Noise in the nervous system. *Nature Reviews Neuroscience,**9*(4), 292–303. 10.1038/nrn2258 18319728 10.1038/nrn2258 PMC2631351

[CR32] Fallon, J., Ward, P. G. D., Parkes, L., Oldham, S., Arnatkevičiūtė, A., Fornito, A., & Fulcher, B. D. (2020). Timescales of spontaneous fMRI fluctuations relate to structural connectivity in the brain. *Network Neuroscience (Cambridge, Mass.)*, *4*(3), 788–806. 10.1162/NETN_A_00151 10.1162/netn_a_00151 PMC7888482 33615091

[CR33] Feldman, D. E. (2009). Synaptic mechanisms for plasticity in neocortex. *Annual Review of Neuroscience*, *32*(Volume 32, 2009), 33–55. 10.1146/ANNUREV.NEURO.051508.135516/CITE/REFWORKS 10.1146/annurev.neuro.051508.135516 PMC3071739 19400721

[CR34] Field, A. (2013). *Discovering statistics using IBM SPSS statistics*. sage.

[CR35] Finley, A. J., Angus, D. J., Knight, E., van Reekum, C. M., Lachman, M. E., Davidson, R. J., & Schaefer, S. M. (2024). Resting EEG Periodic and Aperiodic Components Predict Cognitive Decline Over 10 Years. *The Journal of Neuroscience : The Official Journal of the Society for Neuroscience*. 10.1523/JNEUROSCI.1332-23.2024 10.1523/JNEUROSCI.1332-23.2024 PMC10977020 38373849

[CR36] Fontanini, A., & Katz, D. B. (2008). Behavioral states, network states, and sensory response variability. *Journal of Neurophysiology,**100*(3), 1160–1168. 10.1152/JN.90592.2008/ASSET/IMAGES/LARGE/Z9K0090890410005.JPEG 18614753 10.1152/jn.90592.2008 PMC2544460

[CR37] Fox, J., & Weisberg, S. (2019). *An R Companion to Applied Regression* (Third). Sage. https://socialsciences.mcmaster.ca/jfox/Books/Companion/. Accessed 5 Sept 2024.

[CR38] Gao, R., Peterson, E. J., & Voytek, B. (2017). Inferring synaptic excitation/inhibition balance from field potentials. *NeuroImage,**158*, 70–78. 10.1016/j.neuroimage.2017.06.078 28676297 10.1016/j.neuroimage.2017.06.078

[CR39] Garrett, D. D., Kovacevic, N., McIntosh, A. R., & Grady, C. L. (2011). The importance of being variable. *Journal of Neuroscience,**31*(12), 4496–4503. 10.1523/JNEUROSCI.5641-10.2011 21430150 10.1523/JNEUROSCI.5641-10.2011 PMC3104038

[CR40] Goldberger, A. L., Amaral, L. A. N., Hausdorff, J. M., Ivanov, P. C., Peng, C. K., & Stanley, H. E. (2002). Fractal dynamics in physiology: Alterations with disease and aging. *Proceedings of the National Academy of Sciences of the United States of America,**99*(SUPPL. 1), 2466–2472. 10.1073/PNAS.012579499/ASSET/1F592329-27FB-4DEB-A809-97E3F01232C6/ASSETS/GRAPHIC/PQ0125794008.JPEG 11875196 10.1073/pnas.012579499 PMC128562

[CR41] Harris, K. D., & Thiele, A. (2011). Cortical state and attention. *Nature Reviews Neuroscience, 12*(9), 509–523. 10.1038/nrn3084 10.1038/nrn3084 PMC3324821 21829219

[CR42] Hartig, F., & Lohse, L. (2022). *Package “DHARMa” Residual Diagnostics for Hierarchical (Multi-Level / Mixed) Regression Models* (Issue 1, pp. 1–65). https://cran.r-project.org/web/packages/DHARMa/index.html. Accessed 5 Sept 2024.

[CR43] He, B. J., Zempel, J. M., Snyder, A. Z., & Raichle, M. E. (2010). The temporal structures and functional significance of scale-free brain activity. *Neuron,**66*(3), 353–369. 10.1016/J.NEURON.2010.04.020 20471349 10.1016/j.neuron.2010.04.020 PMC2878725

[CR44] Hickmott, P., & Dinse, H. (2012). Effects of Aging on Properties of the Local Circuit in Rat Primary Somatosensory Cortex (S1) In Vitro. *Cerebral Cortex,**23*(10), 2500–2513. 10.1093/cercor/bhs248 22879353 10.1093/cercor/bhs248

[CR45] Ho, C. S., & Giaschi, D. E. (2009). Low- and high-level first-order random-dot kinematograms: Evidence from fMRI. *Vision Research,**49*(14), 1814–1824. 10.1016/j.visres.2009.04.018 19393261 10.1016/j.visres.2009.04.018

[CR46] Hu, S., Zhang, Z., Zhang, X., Wu, X., & Valdes-Sosa, P. A. (2024). ξ- π: a nonparametric model for neural power spectra decomposition. *IEEE Journal of Biomedical and Health Informatics*, *PP*. 10.1109/JBHI.2024.3364499 10.1109/JBHI.2024.3364499 38335090

[CR47] Hua, T., Li, X., He, L., Zhou, Y., Wang, Y., & Leventhal, A. G. (2006). Functional degradation of visual cortical cells in old cats. *Neurobiology of Aging,**27*(1), 155–162. 10.1016/j.neurobiolaging.2004.11.012 16298251 10.1016/j.neurobiolaging.2004.11.012

[CR48] Hutchinson, C. V., Ledgeway, T., & Allen, H. A. (2014). The ups and downs of global motion perception: A paradoxical advantage for smaller stimuli in the aging visual system. *Frontiers in Aging Neuroscience*, *6*(JUL), 199. 10.3389/FNAGI.2014.00199/BIBTEX 10.3389/fnagi.2014.00199 PMC4126366 25152731

[CR49] Itzcovich, E., Riani, M., & Sannita, W. G. (2017). Stochastic resonance improves vision in the severely impaired. *Scientific Reports*, *7*(1). 10.1038/S41598-017-12906-2 10.1038/s41598-017-12906-2 PMC5634416 28993662

[CR50] Iwasaki, S., Yamamoto, Y., Togo, F., Kinoshita, M., Yoshifuji, Y., Fujimoto, C., & Yamasoba, T. (2014). Noisy vestibular stimulation improves body balance in bilateral vestibulopathy. *Neurology,**82*(11), 969–975. 10.1212/WNL.0000000000000215 24532279 10.1212/WNL.0000000000000215

[CR51] Kanashiro, T., Ocker, G. K., Cohen, M. R., & Doiron, B. (2017). Attentional modulation of neuronal variability in circuit models of cortex. *Elife, 6*, e23978. 10.7554/eLife.23978 10.7554/eLife.23978 PMC5476447 28590902

[CR52] Kilgler, C. F. A., Taghavy, A., & Platt, D. (1993). The event-related P300 potential analysis of cognitive human brain aging: A review. *Gerontology,**39*(5), 280–303. 10.1159/000213544 8314095 10.1159/000213544

[CR53] Kim, Y. J., Grabowecky, M., & Suzuki, S. (2006). Stochastic resonance in binocular rivalry. *Vision Research,**46*(3), 392–406. 10.1016/J.VISRES.2005.08.009 16183099 10.1016/j.visres.2005.08.009

[CR54] Kitajo, K., Doesburg, S. M., Yamanaka, K., Nozaki, D., Ward, L. M., & Yamamoto, Y. (2007). Noise-induced large-scale phase synchronization of human-brain activity associated with behavioural stochastic resonance. *Europhysics Letters,**80*(4), 40009. 10.1209/0295-5075/80/40009

[CR55] Kitajo, K., Nozaki, D., Ward, L. M., & Yamamoto, Y. (2003). Behavioral Stochastic Resonance within the Human Brain. *Physical Review Letters,**90*(21), 4. 10.1103/PHYSREVLETT.90.218103/FIGURES/3/MEDIUM 10.1103/PhysRevLett.90.218103 12786595

[CR56] Kojima, N., Lamsal, B., Matsumoto, N., & Yamashiro, M. (2019). Proposing autotuning image enhancement method using stochastic resonance. *Electronics and Communications in Japan,**102*(4), 35–46. 10.1002/ecj.12160

[CR57] Kumral, D., Şansal, F., Cesnaite, E., Mahjoory, K., Al, E., Gaebler, M., Nikulin, V. V., & Villringer, A. (2020). BOLD and EEG signal variability at rest differently relate to aging in the human brain. *NeuroImage,**207*, 116373. 10.1016/J.NEUROIMAGE.2019.116373 31759114 10.1016/j.neuroimage.2019.116373

[CR58] Kundu, A., & Sarkar, S. (2015). Stochastic resonance in visual sensitivity. *Biological Cybernetics,**109*(2), 241–254. 10.1007/S00422-014-0638-Y 25398687 10.1007/s00422-014-0638-y

[CR59] Lange, K., Kühn, S., & Filevich, E. (2015). "Just Another Tool for Online Studies” (JATOS): An Easy Solution for Setup and Management of Web Servers Supporting Online Studies. *PLoS ONE,**10*(6), e0130834. 10.1371/journal.pone.0130834 26114751 10.1371/journal.pone.0130834 PMC4482716

[CR60] Leventhal, A. G., Wang, Y., Pu, M., Zhou, Y., & Ma, Y. (2003). GABA and its agonists improved visual cortical function in senescent monkeys. *Science,**300*(5620), 812–815. 10.1126/science.1082874 12730605 10.1126/science.1082874

[CR61] Levitt, H. (1971). Transformed Up-Down Methods in Psychoacoustics. *The Journal of the Acoustical Society of America,**49*(2B), 467–477. 10.1121/1.1912375 5541744

[CR62] Li, S.-C., Lindenberger, U., & Sikström, S. (2001). Aging cognition: From neuromodulation to representation. *Trends in Cognitive Sciences,**5*(11), 479–486. 10.1016/S1364-6613(00)01769-1 11684480 10.1016/s1364-6613(00)01769-1

[CR63] Li, S. C., von Oertzen, T., & Lindenberger, U. (2006). A neurocomputational model of stochastic resonance and aging. *Neurocomputing,**69*(13–15), 1553–1560. 10.1016/J.NEUCOM.2005.06.015

[CR64] Lipsitz, L. A., & Goldberger, A. L. (1992). Loss of “Complexity” and Aging: Potential Applications of Fractals and Chaos Theory to Senescence. *JAMA,**267*(13), 1806–1809. 10.1001/JAMA.1992.03480130122036 1482430

[CR65] Makowski, D., Ben-Shachar, M. S., Patil, I., & Lüdecke, D. (2020). Automated results reporting as a practical tool to improve reproducibility and methodological best practices adoption. *CRAN Available Online*: https://Github.Com/Easystats/Report. Accessed 1 Mar 2023.

[CR66] Manwani, A., & Koch, C. (1999). Detecting and Estimating Signals in Noisy Cable Structures, I: Neuronal Noise Sources. *Neural Computation,**11*(8), 1797–1829. 10.1162/089976699300015972 10578033 10.1162/089976699300015972

[CR67] Manyukhina, V. O., Prokofyev, A. O., Obukhova, T. S., Stroganova, T. A., & Orekhova, E. V. (2024). Changes in high-frequency aperiodic 1/f slope and periodic activity reflect post-stimulus functional inhibition in the visual cortex. *Imaging Neuroscience*. 10.1162/IMAG_A_00146 10.1162/imag_a_00146 PMC12247559 40800396

[CR68] Mazerolle, M. J. (2023). *AICcmodavg: Model selection and multimodel inference based on (Q)AIC(c)*. https://cran.r-project.org/package=AICcmodavg. Accessed 5 Sept 2024.

[CR69] McDonnell, M. D., & Abbott, D. (2009). What is stochastic resonance? Definitions, misconceptions, debates, and its relevance to biology. *PLoS Computational Biology,**5*(5), 1–9. 10.1371/journal.pcbi.1000348 10.1371/journal.pcbi.1000348 PMC2660436 19562010

[CR70] Mendez-Balbuena, I., Manjarrez, E., Schulte-Mönting, J., Huethe, F., Tapia, J. A., Hepp-Reymond, M. C., & Kristeva, R. (2012). Improved sensorimotor performance via stochastic resonance. *Journal of Neuroscience,**32*(36), 12612–12618. 10.1523/JNEUROSCI.0680-12.2012 22956850 10.1523/JNEUROSCI.0680-12.2012 PMC6621271

[CR71] Merkin, A., Sghirripa, S., Graetz, L., Smith, A. E., Hordacre, B., Harris, R., Pitcher, J., Semmler, J., Rogasch, N. C., & Goldsworthy, M. (2023). Do age-related differences in aperiodic neural activity explain differences in resting EEG alpha? *Neurobiology of Aging,**121*, 78–87. 10.1016/J.NEUROBIOLAGING.2022.09.003 36379095 10.1016/j.neurobiolaging.2022.09.003

[CR72] Milne, E. (2011). Increased intra-participant variability in children with autistic spectrum disorders: Evidence from single-trial analysis of evoked EEG. *Frontiers in Psychology,**2*, 51. 10.3389/fpsyg.2011.00051 21716921 10.3389/fpsyg.2011.00051 PMC3110871

[CR73] Moss, F., Ward, L. M., & Sannita, W. G. (2004). Stochastic resonance and sensory information processing: A tutorial and review of application. *Clinical Neurophysiology,**115*(2), 267–281. 10.1016/J.CLINPH.2003.09.014 14744566 10.1016/j.clinph.2003.09.014

[CR74] Moss, F., & Wiesenfeld, K. (1995). The benefits of background noise. *Scientific American,**273*(2), 66–69.

[CR75] Mulavara, A. P., Fiedler, M. J., Kofman, I. S., Wood, S. J., Serrador, J. M., Peters, B., Cohen, H. S., Reschke, M. F., & Bloomberg, J. J. (2011). Improving balance function using vestibular stochastic resonance: Optimizing stimulus characteristics. *Experimental Brain Research,**210*(2), 303–312. 10.1007/S00221-011-2633-Z 21442221 10.1007/s00221-011-2633-z

[CR76] Naik, S., Banerjee, A., Bapi, R. S., Deco, G., & Roy, D. (2017). Metastability in Senescence. *Trends in Cognitive Sciences,**21*(7), 509–521. 10.1016/j.tics.2017.04.007 28499740 10.1016/j.tics.2017.04.007

[CR77] Nomi, J. S., Bolt, T. S., ChiemekaEzie, C. E., Uddin, L. Q., & Heller, A. S. (2017). Moment-to-moment BOLD signal variability reflects regional changes in neural flexibility across the lifespan. *Journal of Neuroscience,**37*(22), 5539–5548. 10.1523/JNEUROSCI.3408-16.2017 28473644 10.1523/JNEUROSCI.3408-16.2017 PMC5452342

[CR78] Parvez, C., Mohammed, D., & Neurosci, M. B. (2021). Differential Circuit Mechanisms of Young and Aged Visual Cortex in the Mammalian Brain. *NeuroSci 2021, Vol. 2, Pages 1–26*, *2*(1), 1–26. 10.3390/NEUROSCI2010001

[CR79] Pathania, A., Euler, M. J., Clark, M., Cowan, R. L., Duff, K., & Lohse, K. R. (2022). Resting EEG spectral slopes are associated with age-related differences in information processing speed. *Biological Psychology*, *168*, 2021.02.12.21251655. 10.1016/j.biopsycho.2022.108261 10.1016/j.biopsycho.2022.108261 34999166

[CR80] Pavan, A., Ghin, F., Contillo, A., Milesi, C., Campana, G., & Mather, G. (2019). Modulatory mechanisms underlying high-frequency transcranial random noise stimulation (hf-tRNS): A combined stochastic resonance and equivalent noise approach. *Brain Stimulation,**12*(4), 967–977. 10.1016/j.brs.2019.02.018 30833217 10.1016/j.brs.2019.02.018

[CR81] Pilz, K. S., Miller, L., & Agnew, H. C. (2017). Motion coherence and direction discrimination in healthy aging. *Journal of Vision,**17*(1), 31. 10.1167/17.1.31 28129415 10.1167/17.1.31

[CR82] Pritchard, W. S. (1992). The brain in fractal time: 1/f-like power spectrum scaling of the human electroencephalogram. *The International Journal of Neuroscience,**66*(1–2), 119–129. 10.3109/00207459208999796 1304564 10.3109/00207459208999796

[CR83] R Core Team. (2013). *The R Stats Package*. Cran. https://stat.ethz.ch/R-manual/R-devel/library/stats/html/00Index.html. Accessed 5 Sept 2024.

[CR84] Radulescu, C. I., Doostdar, N., Zabouri, N., Melgosa-Ecenarro, L., Wang, X., Sadeh, S., Pavlidi, P., Airey, J., Kopanitsa, M., Clopath, C., & Barnes, S. J. (2023). Age-related dysregulation of homeostatic control in neuronal microcircuits. *Nature Neuroscience,**26*(12), 2158–2170. 10.1038/s41593-023-01451-z 37919424 10.1038/s41593-023-01451-z PMC10689243

[CR85] Rajananda, S., Lau, H., & Odegaard, B. (2018). A random-dot kinematogram for web-based vision research. *Journal of Open Research Software*, *6*(1). 10.5334/JORS.194

[CR86] Reuter-Lorenz, P. A., & Park, D. C. (2014). How does it STAC up? Revisiting the scaffolding theory of aging and cognition. *Neuropsychology Review,**24*(3), 355–370. 10.1007/s11065-014-9270-9 25143069 10.1007/s11065-014-9270-9 PMC4150993

[CR87] Rozycka, A., & Liguz-Lecznar, M. (2017). The space where aging acts: Focus on the GABAergic synapse. *Aging Cell,**16*(4), 634–643. 10.1111/acel.12605 28497576 10.1111/acel.12605 PMC5506442

[CR88] Rubenstein, J. L. R., & Merzenich, M. M. (2003). Model of autism: Increased ratio of excitation/inhibition in key neural systems. *Genes, Brain and Behavior,**2*(5), 255–267. 10.1034/j.1601-183X.2003.00037.x 14606691 10.1034/j.1601-183x.2003.00037.x PMC6748642

[CR89] Runnova, A. E., Hramov, A. E., Grubov, V. V., Koronovskii, A. A., Kurovskaya, M. K., & Pisarchik, A. N. (2016). Theoretical background and experimental measurements of human brain noise intensity in perception of ambiguous images. *Chaos, Solitons and Fractals,**93*, 201–206. 10.1016/J.CHAOS.2016.11.001

[CR90] Sasaki, H., Todorokihara, M., Ishida, T., Miyachi, J., Kitamura, T., & Aoki, R. (2006). Effect of noise on the contrast detection threshold in visual perception. *Neuroscience Letters,**408*(2), 94–97. 10.1016/J.NEULET.2006.08.054 16996210 10.1016/j.neulet.2006.08.054

[CR91] Scaglione, A., Moxon, K. A., Aguilar, J., & Foffani, G. (2011). Trial-to-trial variability in the responses of neurons carries information about stimulus location in the rat whisker thalamus. *Proceedings of the National Academy of Sciences of the United States of America,**108*(36), 14956–14961. 10.1073/PNAS.1103168108/SUPPL_FILE/PNAS.201103168SI.PDF 21873241 10.1073/pnas.1103168108 PMC3169105

[CR92] Silva, M. F., Harvey, B. M., Jorge, L., Canário, N., Machado, F., Soares, M., & Castelo-Branco, M. (2020). Linked deterioration of early visual perception, function and structure in healthy human aging. *BioRxiv*, 2020.08.05.238014. 10.1101/2020.08.05.238014

[CR93] Simonotto, E., Riani, M., Seife, C., Roberts, M., Twitty, J., & Moss, F. (1997). Visual Perception of Stochastic Resonance. *Physical Review Letters,**78*(6), 1186–1189. 10.1103/PhysRevLett.78.1186

[CR94] Sleimen-Malkoun, R., Temprado, J. J., & Hong, S. L. (2014). Aging induced loss of complexity and dedifferentiation: Consequences for coordination dynamics within and between brain, muscular and behavioral levels. *Frontiers in Aging Neuroscience*, *6*(JUN), 1–1. 10.3389/fnagi.2014.00140 10.3389/fnagi.2014.00140 PMC4073624 25018731

[CR95] Srebro, R., & Malladi, P. (1999). Stochastic resonance of the visually evoked potential. *Physical Review E,**59*(3), 2566–2570. 10.1103/PhysRevE.59.2566

[CR96] Stoffel, M. A., Nakagawa, S., & Schielzeth, H. (2021). partR2: Partitioning R2 in generalized linear mixed models. *PeerJ,**9*, e11414. 10.7717/PEERJ.11414/SUPP-1 34113487 10.7717/peerj.11414 PMC8162244

[CR97] Talyansky, S., & Brinkman, B. A. W. (2021). Dysregulation of excitatory neural firing replicates physiological and functional changes in aging visual cortex. *PLOS Computational Biology,**17*(1), e1008620. 10.1371/JOURNAL.PCBI.1008620 33497380 10.1371/journal.pcbi.1008620 PMC7864437

[CR98] Thuwal, K., Banerjee, A., & Roy, D. (2021). Aperiodic and Periodic Components of Ongoing Oscillatory Brain Dynamics Link Distinct Functional Aspects of Cognition across Adult Lifespan. *ENeuro*, *8*(5). 10.1523/ENEURO.0224-21.2021 10.1523/ENEURO.0224-21.2021 PMC8547598 34544762

[CR99] Tran, T. T., Rolle, C. E., Gazzaley, A., & Voytek, B. (2020). Linked sources of neural noise contribute to age-related cognitive decline. *Journal of Cognitive Neuroscience,**32*(9), 1813–1822. 10.1162/jocn_a_01584 32427069 10.1162/jocn_a_01584 PMC7474516

[CR100] Treviño, M., De la Torre-Valdovinos, B., & Manjarrez, E. (2016). Noise Improves Visual Motion Discrimination via a Stochastic Resonance-Like Phenomenon. *Frontiers in Human Neuroscience,**10*, 572. 10.3389/fnhum.2016.00572 27932960 10.3389/fnhum.2016.00572 PMC5120109

[CR101] Trick, G. L., & Silverman, S. E. (1991). Visual sensitivity to motion: Age-related changes and deficits in senile dementia of the Alzheimer type. *Neurology,**41*(9), 1437–1440. 10.1212/wnl.41.9.1437 1891094 10.1212/wnl.41.9.1437

[CR102] Tripathy, S. P., Shafiullah, S. N., & Cox, M. J. (2012). Influence of Correspondence Noise and Spatial Scaling on the Upper Limit for Spatial Displacement in Fully-Coherent Random-Dot Kinematogram Stimuli. *PLoS ONE,**7*(10), 42995. 10.1371/journal.pone.0042995 10.1371/journal.pone.0042995 PMC3467235 23056172

[CR103] Turrigiano, G. (2011). Too many cooks? Intrinsic and synaptic homeostatic mechanisms in cortical circuit refinement. *Annual Review of Neuroscience,**34*, 89–103. 10.1146/ANNUREV-NEURO-060909-153238 21438687 10.1146/annurev-neuro-060909-153238

[CR104] Uddin, L. Q. (2020). Bring the Noise: Reconceptualizing Spontaneous Neural Activity. *Trends in Cognitive Sciences,**24*(9), 734–746. 10.1016/j.tics.2020.06.003 32600967 10.1016/j.tics.2020.06.003 PMC7429348

[CR105] van Boxtel, J. J. A. (2019). Modeling stochastic resonance in humans: The influence of lapse rate. *Journal of Vision,**19*(13), 19–19. 10.1167/19.13.19 31755904 10.1167/19.13.19

[CR106] van den Brink, R. L., Pfeffer, T., & Donner, T. H. (2019). Brainstem Modulation of Large-Scale Intrinsic Cortical Activity Correlations. *Frontiers in Human Neuroscience,**13*, 486495. 10.3389/FNHUM.2019.00340/BIBTEX 10.3389/fnhum.2019.00340 PMC6794422 31649516

[CR107] van der Groen, O., Tang, M. F., Wenderoth, N., & Mattingley, J. B. (2018). Stochastic resonance enhances the rate of evidence accumulation during combined brain stimulation and perceptual decision-making. *PLoS Computational Biology,**14*(7), e1006301. 10.1371/journal.pcbi.1006301 30020922 10.1371/journal.pcbi.1006301 PMC6066257

[CR108] Varlet, M., Schmidt, R. C., & Richardson, M. J. (2016). Influence of Internal and External Noise on Spontaneous Visuomotor Synchronization. *Journal of Motor Behavior,**48*(2), 122–131. 10.1080/00222895.2015.1050548 26046969 10.1080/00222895.2015.1050548

[CR109] Voytek, B., Kramer, M. A., Case, J., Lepage, K. Q., Tempesta, Z. R., Knight, R. T., & Gazzaley, A. (2015). Age-Related Changes in 1/f Neural Electrophysiological Noise. *Journal of Neuroscience,**35*(38), 13257–13265. 10.1523/JNEUROSCI.2332-14.2015 26400953 10.1523/JNEUROSCI.2332-14.2015 PMC4579381

[CR110] Ward, L. M., Doesburg, S. M., Kitajo, K., MacLean, S. E., & Roggeveen, A. B. (2006). Neural synchrony in stochastic resonance, attention, and consciousness. *Canadian Journal of Experimental Psychology,**60*(4), 319–326. 10.1037/CJEP2006029 17285879 10.1037/cjep2006029

[CR111] Ward, L. M. K., Morison, G., Simmers, A. J., & Shahani, U. (2018). Age-Related Changes in Global Motion Coherence: Conflicting Haemodynamic and Perceptual Responses. *Scientific Reports,**8*(1), 1–11. 10.1038/s41598-018-27803-5 29968729 10.1038/s41598-018-27803-5 PMC6030110

[CR112] Ward, L. M., Neiman, A., & Moss, F. (2002). Stochastic resonance in psychophysics and in animal behavior. *Biological Cybernetics,**87*(2), 91–101. 10.1007/s00422-002-0328-z 12181585 10.1007/s00422-002-0328-z

[CR113] Waschke, L., Kloosterman, N. A., Obleser, J., & Garrett, D. D. (2021). Behavior needs neural variability. *Neuron,**109*(5), 751–766. 10.1016/j.neuron.2021.01.023 33596406 10.1016/j.neuron.2021.01.023

[CR114] Waschke, L., Wöstmann, M., & Obleser, J. (2017). States and traits of neural irregularity in the age-varying human brain. *Scientific Reports,**7*(1), 103432. 10.1038/s41598-017-17766-4 10.1038/s41598-017-17766-4 PMC5727296 29234128

[CR115] Welford, A. T. (1981). Signal, Noise, Performance, and Age. *Human Factors,**23*(1), 97–109. 10.1177/001872088102300109 7228049 10.1177/001872088102300109

[CR116] Wen, H., & Liu, Z. (2016). Separating Fractal and Oscillatory Components in the Power Spectrum of Neurophysiological Signal. *Brain Topography,**29*(1), 13–26. 10.1007/S10548-015-0448-0/FIGURES/9 26318848 10.1007/s10548-015-0448-0 PMC4706469

[CR117] Whitten, T. A., Hughes, A. M., Dickson, C. T., & Caplan, J. B. (2011). A better oscillation detection method robustly extracts EEG rhythms across brain state changes: The human alpha rhythm as a test case. *NeuroImage,**54*(2), 860–874. 10.1016/J.NEUROIMAGE.2010.08.064 20807577 10.1016/j.neuroimage.2010.08.064

[CR118] Wilson, L. E., Castanheira, J. da S., & Baillet, S. (2022). Time-resolved parameterization of aperiodic and periodic brain activity. *ELife*, *11*. 10.7554/eLife.77348 10.7554/eLife.77348 PMC9467511 36094163

[CR119] Yamazaki, H., & Lioumis, P. (2022). Stochastic resonance at early visual cortex during figure orientation discrimination using transcranial magnetic stimulation. *Neuropsychologia,**168*, 108174. 10.1016/J.NEUROPSYCHOLOGIA.2022.108174 35143870 10.1016/j.neuropsychologia.2022.108174

[CR120] Yan, F. F., Hou, F., Lu, H., Yang, J., Chen, L., Wu, Y., Chen, G., & Huang, C. B. (2020). Aging affects gain and internal noise in the visual system. *Scientific Reports*, *10*(1). 10.1038/S41598-020-63053-0 10.1038/s41598-020-63053-0 PMC7174411 32317655

[CR121] Yi, M., Jia, Y., Liu, Q., Li, J., & Zhu, C. (2006). Enhancement of internal-noise coherence resonance by modulation of external noise in a circadian oscillator. *Physical Review E,**73*(4), 41923. 10.1103/PhysRevE.73.041923 10.1103/PhysRevE.73.041923 16711852

[CR122] Zanker, J. M. (1995). Does motion perception follow Weber’s law? *Perception,**24*(4), 363–372. 10.1068/p240363 7675617 10.1068/p240363

[CR123] Zeng, F. G., Fu, Q. J., & Morse, R. (2000). Human hearing enhanced by noise. *Brain Research,**869*(1–2), 251–255. 10.1016/S0006-8993(00)02475-6 10865084 10.1016/s0006-8993(00)02475-6

[CR124] Zhang, L., Zheng, W., Xie, F., & Song, A. (2017). Effect of the correlation between internal noise and external noise on logical stochastic resonance in bistable systems. *Physical Review E,**96*(5), 52203. 10.1103/PhysRevE.96.052203 10.1103/PhysRevE.96.052203 29347692

